# Workers Who Stay at Work Despite Chronic Nonspecific Musculoskeletal Pain: Do They Differ from Workers with Sick Leave?

**DOI:** 10.1007/s10926-012-9360-6

**Published:** 2012-03-28

**Authors:** Haitze J. de Vries, Michiel F. Reneman, Johan W. Groothoff, Jan H. B. Geertzen, Sandra Brouwer

**Affiliations:** 1 Department of Rehabilitation Medicine, Center for Rehabilitation, University Medical Center Groningen, University of Groningen, P.O. Box 30.002, 9750 RA Haren, The Netherlands; 2 Department of Health Sciences, Community and Occupational Medicine, University Medical Center Groningen, University of Groningen, Groningen, The Netherlands

**Keywords:** Staying at work, Vocational rehabilitation, Musculoskeletal disorders, Chronic pain, Work participation

## Abstract

*Purpose* Most workers with chronic nonspecific musculoskeletal pain (CMP) do not take sick leave, nor consult a health care professional or search vocational rehabilitation. Yet, the knowledge of many researchers, clinicians and policy makers is largely based on people with CMP who discontinue work. The aim of this study was to explore characteristics of workers who stay at work despite CMP, and to compare these with sick-listed workers with CMP following vocational rehabilitation. *Methods* The clinical characteristics of workers who stay at work despite CMP (n = 119) and sick-listed workers who follow vocational rehabilitation (n = 122) were described and the differences between these groups were assessed. Logistic regression analysis was used to assess differences between the groups and to determine which variables predicted group status. *Results* Workers who stayed at work despite CMP reported significantly lower levels of fear avoidance (OR = 0.94), pain catastrophizing (OR = 0.93), perceived workload (OR = 0.93), and higher pain acceptance (OR = 1.11), life control (OR = 1.62) and pain self-efficacy (OR = 1.09) compared to sick-listed workers following rehabilitation, even after controlling for confounders. The groups did not differ on physical activity level, active coping and work satisfaction. Group status was predicted best by pain intensity, duration of pain, pain acceptance, perceived workload, mental health, and psychological distress (area under the receiver operating characteristic curve = 0.91, 95% CI = 0.87–0.95). *Conclusions* A wide range of characteristics of workers who stay at work despite CMP were explored. Relevant differences from sick-listed workers with CMP were observed in all domains of the bio-psycho-social model. Six main predictors were identified that best discriminate between both groups.

## Introduction

The reference of many researchers, clinicians and policy makers concerning work and pain is based on people with chronic nonspecific musculoskeletal pain (CMP) who were not longer able to participate in work. However, by far not all workers with CMP become work-disabled [1–3], nor do they consult a health professional [4–6] or search multidisciplinary rehabilitation. Many workers are able to cope with CMP at work and maintain their employment. It is currently unknown on which factors people who stay at work despite CMP (SAW group) differ from people who are on sick leave and referred for rehabilitation (SL-Rehab group).

Most research has focused on sick leave and work disability of people with CMP [7–9]. Several predictors or associations for work disability have been identified, such as fear avoidance [10, 11], catastrophizing [12, 13], de-conditioning [14, 15], pain acceptance [16, 17], emotional distress [18, 19], life control and self-efficacy [20, 21]. Our knowledge about staying at work with CMP, however, is limited. A literature review to identify factors that promote staying at work in workers with CMP revealed only 7 studies [22]. It was concluded that perceived physical disability and emotional distress are associated with staying at work (low level of evidence). Most studies investigating work participation in workers with CMP focused on absent or disabled workers and did not report on the successful counterpart that remained at work. To learn more about this large but relative “unknown” group, the project Working with Pain was conceived. In this project, staying at work was defined as sustained work participation despite CMP, with a maximum of 5% sick leave over a period of 12 months for CMP reasons. Because this group can be considered as the long-term goal of vocational rehabilitation, we expected that lessons can be learned from these successful workers. Specific attention to this SAW group may broaden our views on chronic pain and work participation. Factors associated with sick leave or disability may also explain why some people succeed to stay at work, where others fail [13, 23]. The theory of fear avoidance describes how people with CMP develop catastrophizing thoughts and inactivity, then become deconditioned, which explains why they develop chronic pain and ultimately are susceptible for work disability. “Acceptance and Commitment Therapy” postulates that people may achieve better adjustment to CMP by learning to reduce avoidance and other attempts to control pain and choosing to direct their efforts on important life-values such as work [16]. People with high levels of stress may easily get trapped in a vicious circle, in which pain and distress reinforce one another. Relief of emotional distress may help people to stay at work [19, 24]. The person’s belief of having control over events may determine the behavior to fulfill its goals [25]; high feelings of control may initiate actions to enhance workability and staying at work. Self-efficacy beliefs determine “how much effort people will expend and how long they will persist in the face of obstacles and aversive experiences” [26–28]. Vocational rehabilitation operates at the interface of work and health care, where a bio-psycho-social approach is required to offer appropriate care. Therefore, a range of variables and corresponding measures were investigated in this study, sufficient to cover most essential domains for work participation: demographic, physical, psychological and work characteristics. It was assumed that if modifiable factors that associate with staying at work are known, it would give new insights for the development of effective vocational rehabilitation programs. Moreover, the knowledge gathered in this study might provide data towards a new reference for clinicians and researchers working in rehabilitation and occupational medicine.

The first aim of this study was to describe physical, psychological and work characteristics of workers in a SAW group. The second aim was to compare these characteristics with a SL-Rehab group and healthy working controls. Our hypotheses regarding the SAW and SL-Rehab group were that compared to the SL-Rehab group, workers in the SAW group report: higher levels of daily activity (hypothesis 1; H1); lower levels of fear avoidance beliefs about physical activity (H2) and pain catastrophizing (H3); higher pain acceptance (H4); lower psychological distress (H5); better life control (H6) and self-efficacy (H7); better active coping (H8); lower perceived physical workload (H9) and higher work satisfaction (H10). Ultimately, the third aim was to examine on which variables the two groups can be distinguished the best.

## Methods

### Design

In a cross-sectional design the characteristics of workers with CMP in a SAW group and SL-Rehab group were measured in order to compare both groups.

### Subjects

Eligible participants of the SAW group were recruited from May 2009 to December 2010 by announcements in newspapers, and websites of national patient associations of whiplash and fibromyalgia. It was made clear that they participated in scientific research and that no treatment or advice would be provided. A compensation of €50 and traveling compensation was offered for participation. Inclusion criteria were: diagnosed as CMP (pain in back, neck, shoulder, extremities or disorders such as widespread pain, fibromyalgia and whiplash) without known underlying specific medical cause (e.g., infection, neoplasm, metastasis, osteoporosis, rheumatoid arthritis, fracture, neurological disorders, and serious spinal pathology); duration of pain was longer than 6 months; age 20–60 years; paid work for 20 h or more during the 12 months before participation in the study. Exclusion criteria in this study were the following: relevant co-morbidities with severe negative consequences for physical and/or mental functioning (for example severe psychiatric disease or addiction to drugs), pregnancy, and insufficient knowledge of the Dutch language. Participants must have sustained work participation despite CMP, operationally defined as a maximum of 5% sick leave ascribed to CMP over a period of 12 months (which is around the average rate of sickness absence in The Netherlands) [29, 30]. Participants did not seek help in a Rehabilitation Center in the year prior to participation.

Workers in the SL-Rehab group were consecutively included from July 2009 to March 2011. The SL-Rehab group was referred for vocational rehabilitation, a multidisciplinary approach that is provided to individuals of working age with health-related impairments, limitations, or restrictions with work functioning and whose primary aim is to optimize work participation [31]. Inclusion and exclusion criteria for the SL-Rehab group were the same as for the SAW group, except for absence at work caused by the pain in the SL-Rehab group was higher than 5% in the year prior to participation.

Sample size was determined by the amount of independent variables we intended to include into a logistic model. A minimum of 10 subjects per independent variable has been recommended [32]. Because we estimated to use 20 predicting variables in the model, a total sample size of at least 200 was needed.

In literature, norm scores or reference data of healthy controls were available for most of the used measures in our study. These reference data were obtained from working healthy controls, aged between 20 and 60 years.

### Procedures

To diagnose the type of pain and the existence of co-morbidities, all participants from both groups received medical examination performed by a physiatrist. All participants completed questionnaires assessing demographic data and physical, psychological and work characteristics. The SL-Rehab group completed the work related questionnaires in relation to their most recent job experiences. Measures were taken prior to the rehabilitation program. Most of the questionnaires are used in usual care of patients in rehabilitation. The study was approved by the Medical Ethical Committee of the University Medical Center Groningen. All participants signed informed consent.

### Measures

#### Demographic Characteristics

Demographic characteristics were gathered by a questionnaire constructed by Rehabilitation Development Centers in the Netherlands [33].

#### Physical Characteristics

##### Pain intensity

Current pain intensity was measured by the 11-point Numeric Rating Scale (NRS), ranging from 0 (no pain) to 10 (worst imaginable pain). Validity and utility of the NRS is sufficient [34, 35].

##### Disability

The Pain Disability Index (PDI) was used to measure the degree to which chronic pain interferes with daily activities (self perceived disability). The PDI is a 7-item inventory, each item score ranging from 0 (no interference) to 10 (total interference). The reliability and validity of the PDI is sufficient [36, 37]. Higher scores reflect higher interference of pain with daily activities. Reference data were obtained from a German population [38].

##### Health

The Dutch version of the RAND 36-item Health survey (RAND-36) was used to measure physical health [39]. The subscales physical functioning, role limitations arising from physical health problems, pain, and general health perception were merged into the Physical Component Summary [40]. Scores range from 0 to 100, and higher scores reflect better perceived physical health. The Dutch version of the RAND 36-items is a reliable, valid and sensitive instrument [39]. Reference data were obtained from a Dutch population [39] and from a Dutch reference sample of healthy workers [41].

##### Activity level

The Baecke Physical Activity Questionnaire (BPAQ; 16 items) was used to assess the total daily physical activity level of participants, reflected by 3 subscales work, sports and (non-sport) leisure time. Higher scores reflect higher perceived activity level. The BPAQ is presented as a valid and reliable instrument [42, 43]. Reference data were obtained from a Dutch reference sample of healthy workers [41].

#### Psychological Characteristics

##### Mental health

The RAND-36 was used to measure mental health. The subscales social functioning, role limitations caused by emotional problems, mental health, and vitality were merged into the Mental Component Summary [40]. Scores range from 0 to 100, and higher scores reflect higher perceived mental health.

The Symptom Checklist-90-Revised (SCL-90-R; 90 items) was used to measure psychosocial distress. The total score, the Global Severity Index (GSI), is reflected by the sum of all sub scores as a global measure of psychological distress. Higher scores reflect higher perceived psychological distress. Reliability and validity of the SCL-90-R are good [44, 45]. Reference data were obtained from a Dutch population [45].

##### Acceptance

Pain acceptance was assessed using the Chronic Pain Acceptance Questionnaire (CPAQ; 20 items) [46, 47], consisting of two subscales: Activity Engagement (participation in daily activities while acknowledging the presence of pain) and Pain Willingness (the degree to which pain is allowed in experience without efforts to avoid or control it). Higher scores reflect higher perceived acceptance of pain. Validity and reliability of the CPAQ are reasonable [48–50]. Reference data were not available.

##### Avoidance

Fear avoidance beliefs about physical activity and (re)injury was measured with the Dutch version of the Tampa Scale of Kinesiophobia (TSK; 17 items) [51, 52]. Higher scores reflect higher perceived fear of physical activity. Reliability and validity of the Dutch version are good [52, 53]. Reference data of a healthy working group were not available.

##### Self-efficacy

Pain self-efficacy was measured by the Dutch version of the Pain Self Efficacy Questionnaire (PSEQ; 10 items). Each item is rated by selecting a number on a 7-point scale, scores ranging from 0 (“not at all confident”) to 6 (“completely confident”). Higher scores reflect stronger self-efficacy beliefs. Self-efficacy beliefs for people experiencing chronic pain incorporate not just the expectation that a person could perform a particular behavior or task, but also their confidence in being able to do it despite their pain [54]. The PSEQ has strong psychometric properties and high reliability and validity [28].

##### Catastrophizing

Pain catastrophizing was measured by the Dutch version of the Pain Catastrophizing Scale (PCS; 13 items) [55, 56]. Higher scores reflect stronger experienced thoughts and feelings of participants while they are in pain. The PCS showed to be valid and highly reliable [56–58]. Reference data were obtained from a Dutch community sample without pain [59].

Coping reactions were measured by the Utrecht’s Coping List (UCL; 47 items), distinguished by the following subscales: active coping, palliative reaction, avoidance, social support, passive coping, expression of emotions and coping self statements. Higher scores reflect higher levels of coping reactions. The UCL is validated for patients with chronic pain [60]. Reliability and validity are moderate to good [61]. Reference data were obtained from a Dutch population [61].

Interference of pain in daily life: The Dutch version of the West-Haven Yale Multidimensional Pain Inventory (MPI-DV; 21 items) was used to assess the subjects’ level of life control (incorporating the ability to solve problems and feelings of personal mastery and competence); mood (including ratings of depressed mood, irritability and tension); support received from spouse; and responses of significant others to their pain behavior (punishing, solicitous, and distracting responses). Higher scores reflect stronger feelings of life control, better mood, higher perceived support and more responses of significant others. The reliability and validity of the MPI are good [62, 63].

#### Work Characteristics

Vocational sector, perceived workability, sick leave during previous 12 months, and expectation to fulfill future work were assessed with the Work Ability Index (WAI). The reliability and validity of the WAI are acceptable [64, 65].

Presenteeism was assessed with the World Health Organization’s Health and Work Performance Questionnaire (HPQ). Presenteeism was conceptualized as a measure of actual performance in relation to possible performance, scored as percent of performance on a 0–10 response scale, where 0 represents a total lack of performance and 10 no lack of performance during time of the job. The HPQ is a reliable and valid measure [66, 67].

Work pace, emotional workload, relation with colleagues or supervisor, work satisfaction, and need for recovery were assessed by the Dutch questionnaire on the Perception and Evaluation of Work (Dutch abbreviation: VBBA) [68]. Subscale scores range between 0 and 100; higher scores indicate more unfavorable situations. The reliability and unidimensionality of all scales of the VBBA were considered satisfactory [68]. Reference data were obtained from a Dutch reference sample of healthy workers [41].

The work physical demand category was assessed by the researcher according to the Dictionary of Occupational Titles (DOT). Within the DOT, occupations are classified into 5 categories of physical workload, based on intensity and duration of lifting or carrying needed for the job: sedentary, light, medium, heavy, very heavy [69].

Self reported physical work load was assessed with the Dutch Musculoskeletal Questionnaire (Dutch abbreviation: VBA; 21 items) [70]. Exposure to carrying, lifting, bending, reaching, turning, use of forces, repetitive tasks, and prolonged (inconvenient) postures is measured, reflected in a sum score ranging from 21 to 84. Higher scores reflect a higher physical workload. Reference data were obtained from a Dutch reference sample of healthy workers [41].

### Statistical Analysis

All statistical analyses were performed using SPSS for Windows, version 18.0.3. Missing data in questionnaires were addressed by adding the calculated average of a scale or questionnaire, conform questionnaire recommendations. To create a “profile” of the SAW group, the two groups were first compared on the basis of demographic, physical, psychological and work characteristics. Group differences between the SAW group and SL-Rehab group were analyzed by independent samples *T* tests (continuous measure and normally distributed), or Mann–Whitney *U* tests and Chi-square tests (data not distributed normally). Cohen’s d effect sizes (ES) were calculated to assess the clinical relevance of differences. ES was defined as the difference between two mean scores expressed in standard deviation (sd) units: (x_1_ − x_2_)/σ_pooled_, where σ_pooled_ = √(sd_1_^2^ + sd_2_^2^/2). When comparing group averages, an ES <0.2 was considered as trivial, from 0.2 to 0.49 as small, from 0.5 to 0.79 as medium, and ≥0.8 as large [71]. We considered an ES ≥0.5 as clinically relevant [71, 72].

To test the hypotheses, logistic regression analyses were performed to analyze the contribution of the variables to the dependent variable group status, while controlling for potential confounding variables such as age [73], gender [74], educational level [75, 76], diagnose group, duration of pain, pain intensity [77, 78], and DOT category [69]. Because of the large number of 10 variables, the Bonferroni correction could have been applied to reduce the chance on type-I error, resulting in a *P*-value of 0.005 (0.05/10 variables), which would have reduced the number of variables significantly associated with group status. However, to reduce the chance on type-II errors, we decided not to use the Bonferroni correction.

Stepwise backwards logistic regression was used to assess which of the variables best predicted group status. Based on previous research and theory we selected candidate predictors for group status and entered these in the model. We used a preselected significance value *P* < 0.10 as a criterion for removal from the backwards stepwise analysis to reduce the chance of type-II errors [79]. The Hosmer and Lemeshow test was used to assess how well the chosen model fits the data. To evaluate the ability of the model to discriminate between workers in the SAW and SL-Rehab group, the area under the receiver operating characteristic curve (AUC) was calculated. An AUC of 0.50 indicates no, 0.70–0.80 acceptable, and >0.80 excellent discrimination [79].

## Results

A total of 119 participants were included in the SAW group and 122 in the SL-Rehab group; total sample size was 241. Seven potential participants in the SAW group were not included in the study because of heart disease (2), high blood pressure (2), neurological disorder (1), radiculopathy (1) and co-morbidity (1). Various potential participants registered for the study, but were not allowed to participate because of age >60 years (20), specific medical cause such as rheumatoid arthritis (48), unpaid job (11), employment less than 20 h (14), or more than 5% sick leave (15).

### Description of SAW and SL-Rehab Group

**Fig. 1:**
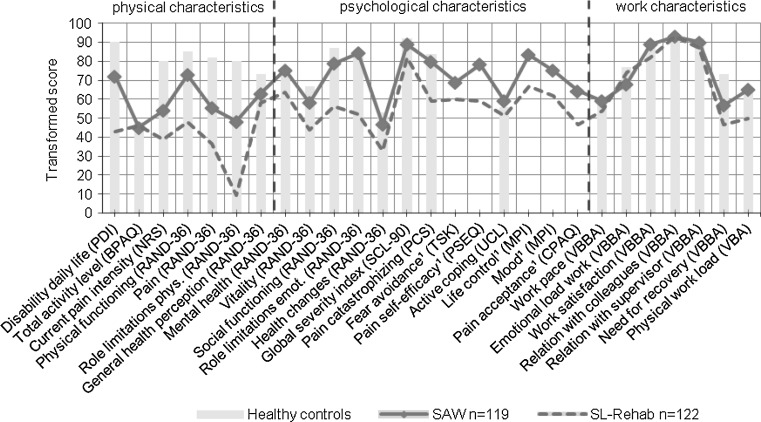
A comparison of the SAW group, SL-Rehab group, and healthy working controls. The y-axis represents transformed scores on a standardized 0–100 scale, in which higher scores represent more favorable situations. The x-axis shows all variables. No norm scores of healthy controls were retrieved for variables indicated with ¹

Demographic, physical, psychological and work characteristics of both groups are presented in Table 1. In Fig. 1 the average scores of the SAW group and SL-Rehab group are presented, supplemented with norm scores from healthy controls. To allow presentation of all variables simultaneously, all scores were transformed to a score ranging from 0 to 100, where higher scores represent a more favorable situation. Transformed scores were only used for Fig. 1 and not in the statistical analyses. In the *demographic characteristics* category, compared to the SL-Rehab group, people in the SAW group had higher age and educational level, longer duration of pain and lower use of pain medication. Major differences between both groups were observed on *physical characteristics*, such as perceived pain and disability, physical functioning and physical role limitations. Moreover, workers in the SL-Rehab group perceive more pain, mental and social limitations, and score detrimental on most *psychological* measures. Both groups scored similar on *work characteristics* such as work pace, emotional load at work, relation with colleagues and supervisor, work satisfaction and need for recovery, but workers in the SAW group reported lower physical activity at work and perceived lower physical workload, which was consistent with the higher percentage of subjects working in a higher DOT-category in the SL-Rehab group.

**Table 1:** Description of demographic, physical, psychological and work characteristics of the SAW and SL-Rehab group

Instrument	Unit or scale	SAW (n = 119) Mean (sd)	SL-Rehab (n = 122) Mean (sd)	n	Effect size	*P*-value
*Demographic characteristics*						
Age	Years	51 (44–54)	39 (32–48)	122		0.001^r^
Gender male	%	40.3	46.0	122		0.380^q^
Married/co-habitation	%	90	72	122		0.001^q^
Educational level	%			106		0.001^q^
Low		11	30			
Medium		56	49			
High		33	21			
Diagnosis region	%			122		0.006^q^
Back		53	66			
Neck/shoulders		13	18			
Fibromyalgia		23	7			
Other^a^		11	9			
Duration of pain	%			96		0.001^q^
1–2 years		8.4	34.4			
2–5 years		10.9	17.8			
>5 years		80.7	47.8			
Pain medication (yes)	%	39.5	85.1	73		0.001^q^
Frequency use pain medication	%			51		0.001^q^
≤3/month		65	10			
1–6/week		21	13			
≥1/day		14	77			
*Physical characteristics*						
NRS current pain^b^	0–10	4.6 (2.1)	6.1 (1.9)	114	0.8	0.001
NRS worst pain	0–10	6.9 (1.8)	8.0 (1.4)	88	0.7	0.001
PDI^c^	0–70	19.9 (11.1)	39.2 (11.2)	92	1.7	0.001
RAND 36^d^						
Physical functioning	0–100	72.8 (17.9)	48.0 (19.8)		1.3	0.001
Role limitations (physical)	0–100	50 (0–100)	0 (0–0)	93	1.2	0.001^r^
Pain	0–100	55.4 (15.5)	36.6 (17.0)	93	1.2	0.001
General health perception	0–100	62.9 (17.7)	58.2 (18.9)	93	0.3	0.072
Health changes	0–100	46.6 (18.7)	32.8 (24.8)	93	0.6	0.001
Physical component summary	0–100	59.8 (17.0)	38.5 (12.7)	93	1.4	0.001
BPAQ^e^						
Work	1–5	2.7 (0.6)	3.2 (0.6)	116	0.8	0.001
Sport	1–5	2.6 (0.8)	2.3 (0.6)	118	0.4	0.004
Leisure time	1–5	3.1 (0.6)	3.0 (0.6)	118	0.2	0.108
Total activity level	3–15	8.4 (1.2)	8.5 (1.1)	116	0.1	0.625
*Psychological characteristics*						
RAND 36^d^						
Social functioning	0–100	78.7 (18.8)	56.2 (24.3)	93	1.0	0.001
Role limitations (emotional)	0–100	100 (100–100)	67 (0–100)	93	0.8	0.001^r^
Mental health	0–100	75.4 (16.4)	63.6 (16.2)	93	0.7	0.001
Vitality	0–100	58.1 (18.3)	43.9 (16.9)	93	0.8	0.001
Mental component summary	0–100	74.1 (17.0)	54.6 (20.2)	93	1.0	0.001
SCL90-R^f^						
Anxiety	10–50	12 (10–14)	14 (12–17)	108	0.5	0.001^r^
Phobic anxiety	7–35	7 (7–8)	7 (7–9)	108	0.4	0.050^r^
Depression	16–80	20 (17–25)	26 (21–35)	108	0.6	0.001^r^
Somatization	12–60	20.9 (5.7)	25.5 (6.3)	108	0.8	0.001
Obsessive–compulsive	9–45	14.8 (4.3)	20.8 (11.3)	108	0.7	0.001
Interpersonal sensitivity	18–90	22 (19–28)	24 (20–31)	108	0.2	0.189^r^
Hostility	6–30	7 (6–7)	8 (7–9)	108	0.6	0.001^r^
Sleep disturbance	3–15	5 (4–7)	7 (5–11)	108	0.5	0.001^r^
Psychoticism	9–45	10 (9–12)	12 (10–14)	108	0.4	0.003^r^
Global severity index	90–450	118 (105–141)	142 (123–177)	108	0.7	0.001^r^
CPAQ^g^						
Activity engagement	0–66	43.5 (7.2)	34.6 (9.6)	118	1.0	0.001
Pain willingness	0–54	28.7 (7.5)	21.4 (7.1)	118	1.0	0.001
Total score	0–120	72.2 (11.7)	56.4 (13.1)	118	1.3	0.001
TSK^h^	17–68	33.0 (7.2)	37.2 (8.1)	107	0.5	0.001
PSEQ self efficacy^i^	0–60	46.9 (8.5)	35.5 (12.0)	121	1.1	0.001
PCS^j^	0–52	10.5 (8.6)	21.6 (10.4)	77	1.2	0.001
Rumination	0–16	4.7 (3.6)	8.2 (3.9)	77	0.9	0.001
Magnification	0–12	1.2 (1.6)	3.1 (2.4)	77	0.9	0.001
Helplessness	0–24	4.5 (4.1)	10.1 (4.8)	77	1.3	0.001
UCL^k^						
Active coping	7–28	19.3 (3.4)	17.7 (3.4)	109	0.5	0.001
Palliative reaction	8–32	17.7 (3.4)	17.6 (3.7)	109	0.0	0.768
Avoidance	8–32	16.2 (3.4)	15.8 (3.2)	109	0.1	0.305
Social support	6–24	13.1 (3.6)	12.8 (3.4)	109	0.1	0.508
Passive coping	7–28	10.9 (3.0)	12.0 (3.1)	109	0.4	0.012
Expression of emotions	3–12	5.7 (1.5)	5.3 (1.6)	109	0.3	0.049
Coping self statements	5–20	12.6 (2.7)	11.9 (2.6)	109	0.3	0.042
MPI^l^						
Life control	0–6	5.0 (4.7–5.7)	4.0 (3.0–5.0)	119	0.9	0.001^r^
Mood	0–6	4.7 (3.7–5.3)	3.7 (2.7–5.0)	120	0.6	0.001^r^
Support	0–6	4.0 (3.0–4.9)	5.0 (4.0–5.3)	100	0.6	0.001^r^
Punishing responses	0–6	1.0 (0.3–1.7)	1.3 (0.3–2.7)	100	0.3	0.029^r^
Solicitous responses	0–6	2.3 (1.1)	2.8 (1.0)	100	0.5	0.001
Distracting responses	0–6	2.4 (1.4)	2.9 (1.2)	99	0.4	0.012
*Work characteristics*						
Expected to work last week	Hours	31.5 (7.8)	35.0 (11.1)	122	0.4	0.007
Actually worked last week	Hours	32.5 (10.4)	11.3 (13.8)	113	1.7	0.001
HPQ presenteeism^m^	0–100	76.9 (11.1)	46.7 (29.5)	89	1.4	0.001
HPQ relative presenteeism	0.25–2	1.1 (0.3)	0.75 (0.4)	85	1.0	0.001
Employment	%			114		0.260^q^
Part-time		49.6	42.2			
Full-time		50.4	57.8			
Sick leave	%			122		0.001^q^
<5%		100	0			
5–20%		0	16.5			
21–50%		0	20			
>50%		0	63.5			
Vocational sector	%			115		
Industry		8	13			
Construction		1	8			
Trade		9	18			
Transport		4	5			
Commercial services		9	7			
Education		13	7			
Health care		34	25			
Public administration		13	7			
Agriculture		4	4			
Other		5	6			
Work demands						
Physical demand category work				122		0.007^q^
DOT category 1^n^	%	35	20			
DOT category 2	%	35	33			
DOT category 3	%	24	29			
DOT category 4	%	6	18			
VBBA^o^						
Work pace	0–100	41.3 (13.9)	45.8 (15.2)	111	0.3	0.023
Emotional load	0–100	31.9 (15.1)	25.8 (15.1)	111	0.4	0.003
Relation with colleagues	0–100	0 (0–11)	0 (0–11)	109	0.0	0.560^r^
Relation with supervisor	0–100	0 (0–11)	0 (0–11)	106	0.1	0.710^r^
Work satisfaction	0–100	0 (0–11)	0 (0–22)	110	0.3	0.024^r^
Need for recovery	0-100	45 (18–73)	64 (18–82)	109	0.3	0.020^r^
VBA^p^	21–84	43.1 (10.4)	52.5 (12.3)	112	0.8	0.001

^a^Pain of extremity, cervical-brachial syndrome, generalized pain, ^b^ Numeric Rating Scale (0 = no pain, 10 = worst possible pain), ^c ^Pain Disability Index, ^d ^RAND 36-item Health Survey, ^e ^Baecke Physical Activity Questionnaire, ^f^ Symptom Checklist 90-R, ^g^ Chronic Pain Acceptance Questionnaire, ^h^ Tampa Scale for Kinesiophobia, ^i^ Pain Self Efficacy Questionnaire, ^j^ Pain Catastrophizing Scale, ^k^ Utrecht’s Coping List, ^l^ Multidimensional Pain Inventory, ^m^ Health and Work Performance Questionnaire, ^n^ Dictionary of Occupational Titles; 1 = sedentary; 2 = light; 3 = medium; 4 = heavy/very heavy work, ^o^ Questionnaire on the Perception and Evaluation of Work, ^p^ Dutch Musculoskeletal Questionnaire, ^q^ Chi-squared test (*χ* ^2^-test), ^r^ Mann-Whitney *U* test, outlined in the table with median (25–75% inter-quartile range)

The largest differences with the healthy controls were found in the physical characteristics category; scores in the psychological and work categories are generally similar with the SAW group.

### Hypotheses Tested

In Table 2 the results of the hypothesis testing are presented. In six variables a significant association with group status was observed: fear avoidance beliefs about physical activity (OR 0.94, *P* = 0.028), pain catastrophizing (OR 0.93, *P* = 0.005), pain acceptance (OR 1.11, *P* = 0.001), pain self-efficacy (OR 1.09, *P* = 0.001), life control (OR 1.62, *P* = 0.012), and perceived physical workload (OR 0.93, *P* = 0.003), even after adjusting for potential confounders. Psychological distress was almost significantly associated with group status. No association with group status was observed for activity level, active coping and work satisfaction.

**Table 2:** Hypotheses (H) tested by logistic regression, adjusted for potential confounders, with group status as dependent variable

Instrument	Hypothesis	n	B	*P*-value	Exp(B)	95% CI Exp(B)
Physical characteristics						
H1: Activity level^a^	SAW > SL-Rehab	193	−0.10	0.597	0.91	[0.64–1.30]
Psychological characteristics						
H2: Fear avoidance^b^	SAW < SL-Rehab	190	−0.06	0.028*	0.94	[0.90–0.99]
H3: Pain catastrophizing^c^	SAW < SL-Rehab	165	−0.07	0.005*	0.93	[0.88–0.98]
H4: Pain acceptance^d^	SAW > SL-Rehab	196	0.10	0.001*	1.11	[1.06–1.16]
H5: Psychological distress^e^	SAW < SL-Rehab	190	−0.01	0.082	0.99	[0.98–1.00]
H7: Pain self efficacy^f^	SAW > SL-Rehab	198	0.09	0.001*	1.09	[1.05–1.14]
H6: Life control^g^	SAW > SL-Rehab	196	0.48	0.012*	1.62	[1.11–2.36]
H8: Active coping^h^	SAW > SL-Rehab	191	0.04	0.490	1.04	[0.92–1.18]
Work characteristics						
H9: Work satisfaction^i^	SAW > SL-Rehab	190	−0.00	0.639	1.00	[0.98–1.01]
H10: Physical workload^j^	SAW < SL-Rehab	192	−0.07	0.003*	0.93	[0.89–0.98]

Exp(B) >1 indicated a higher chance to be in the SAW group

* Significant difference, *P* < 0.05

^a^Baecke Physical Activity Questionnaire, ^b^ Tampa Scale for Kinesiophobia, ^c^ Pain Catastrophizing Scale, ^d^ Chronic Pain Acceptance Questionnaire, ^e^ Symptom Checklist R-90, ^f^ Pain Self Efficacy Questionnaire, ^g^ Multidimensional Pain Inventory, ^h^ Utrecht’s Coping List, ^i^ Questionnaire on the Perception and Evaluation of Work, ^j^ Dutch Musculoskeletal Questionnaire

### Discriminating Between SAW and SL-Rehab Group

In Table 3 the results of the backwards stepwise logistic regression analysis are presented. Within this regression model, group status was best discriminated by pain intensity, duration of pain, pain acceptance, perceived workload, mental health, and psychological distress. The Hosmer and Lemeshow test supported our model (χ^2^ = 6.80, *P* = 0.56). The model showed excellent ability to discriminate between the SAW and SL-Rehab group (AUC = 0.91, 95% CI = 0.87–0.95). If the value of the pain intensity scale raises one unit (scale 0–10), the odds of a person to be in the SAW group decrease 1.8 times. When pain duration is longer than 5 years, the odds to be in the SAW group increase 6.4 times. A higher score of one unit on pain acceptance (scale 0–120), mental health (scale 0–100) or psychological distress (scale 90–450) increased the odds to stay at work (OR 1.08, 1.07 and 1.02), while a higher score of one unit on perceived workload (scale 21–84) reduced the odds to stay at work (OR 1.10).

**Table 3:** Results of the logistic regression analysis, with group status as dependent variable

Predictor	B	SE	*P*-value	Exp(B)	95% CI Exp(B)
Physical characteristics					
Pain intensity (1 point higher)	−0.61	0.17	0.001	0.55	[0.39–0.76]
Pain duration (>5 years)	1.86	0.68	0.006	6.40	[1.70–24.00]
Psychological characteristics					
Pain acceptance (1 point higher)	0.08	0.02	0.002	1.08	[1.03–1.14]
Mental health (1 point higher)	0.07	0.02	0.001	1.07	[1.03–1.12]
Psychological distress (1 point higher)	0.02	0.01	0.036	1.02	[1.00–1.04]
Work characteristics					
Perceived workload (1 point higher)	−0.10	0.03	0.002	0.91	[0.85–0.97]

Exp(B) >1 indicated a higher chance to be in the SAW group

χ^2^ = 82.9 (degrees of freedom = 6, n = 151), *P* < 0.001

## Discussion

### Main Findings

The aim of this study was to describe and compare the differences of a SAW group and a SL-Rehab group on physical, psychological and work characteristics. An extended profile of this relative unknown SAW group was presented (Table 1) and a crude comparison with the SL-Rehab group and healthy controls was made (Table 1, Fig. 1). Based on theoretical grounds we hypothesized to identify several differences between the SAW and SL-Rehab group. Significant differences were found for fear avoidance, pain catastrophizing, pain acceptance, pain self-efficacy beliefs, life control and perceived physical workload. The SAW and SL-Rehab group scored similar on activity level, active coping and work satisfaction. Both groups were best discriminated by pain intensity, pain duration, pain acceptance, mental health, psychological distress and perceived workload.

Contrary to the present study, in a systematic review on factors promoting staying at work in workers with CMP, pain catastrophizing was consistently not associated with staying at work [22]. Although different questionnaires were used to measure pain catastrophizing, a plausible explanation for this contradictory observation is unavailable. Pain acceptance has been observed to be associated with better work status [16]. The higher level of pain acceptance experienced by workers in the SAW group means that they participated more in daily activities while acknowledging the presence of pain, and were better able to allow pain in experiences without efforts to avoid or control it. This was not conflicting with the detected higher feelings of life control in our SAW group: paradoxically, when pain control becomes less important, the feeling to have control over life increases. Some people believe that once their pain is solved, they regain the ability to fulfill their work demands. Because these people “rely on the healthcare system and still seek for a medical solution for their pain”, they have decreased power of life control [80].

Workers in the SAW group reported significantly higher pain self-efficacy beliefs compared to sick-listed workers in the SL-Rehab group. Having high self-efficacy beliefs can be considered as a prerequisite for behavior promoting staying at work, such as: raising adjustment latitude, changing pain-coping strategies, organizing modifications and conditions at work, finding access to healthcare services, and asking for support [21, 81]. Many patients with CMP have resistance to behavioral changes or a lack of self-management skills to make that change. Vocational rehabilitation to promote staying at work in people with CMP should consider to target pain self-efficacy.

A systematic review on factors promoting staying at work in people with CMP concluded that low perceived physical disability and low emotional distress were associated with staying at work [22]. This was confirmed in the present study, where large differences were observed on these variables between the groups. Because we selected two groups based on work status and rehabilitation status, it was not surprising that the groups differed on perceived disability. It was also expected that the groups would differ on activity level, however, no difference was observed. The considerable difference on perceived disability between the two groups, while having the same activity level, is remarkable. Even compared with healthy working controls the activity level of workers with CMP, whether sick listed or not, did not differ. This result does not support the assumption of activating to promote returning to work, or activating sports at work for remaining at work, which is often postulated in literature [82, 83]. Simply activating patients with CMP in rehabilitation programs to promote sustained work participation or return to work may be reconsidered, because the working mechanism is unknown, and it may be only effective for subgroups [84]. Coping strategy was not associated with group status. In an interview study on staying at work, participants judged their coping style as an important success factor to stay at work. It appeared that opposite coping strategies (e.g., medication use can be viewed both as an active and a passive coping strategy) could lead to the similar results [81].

People in the SAW group were on average almost 10 years older. This might be the consequence of the selection process; participation into the study was probably more attractive for older people. In addition, the “healthy worker” effect may have resulted in younger workers admitted for rehabilitation, reducing the age in the SL-Rehab group. Older workers, who often had longer duration of pain, may have had more time to re-organize their lives and probably better learned to accept the pain. In another study was observed that older persons were less likely to be out off work due to pain [16] and a few studies observed that age was not associated with staying at work [85–87].

Work factors are frequently associated with sick-leave and work disability [13, 88, 89]. In our study physical factors at work, such as perceived physical workload, were stronger associated with staying at work than psycho-social factors, which is consistent with other research [90]. Workers with strenuous jobs may sooner experience problems to stay at work with CMP. Vocational rehabilitation should improve the functional capacity of these workers, or investigate possibilities for workplace adjustments.

### Discriminating Between SAW and SL-Rehab Group

In the stepwise logistic regression model, being in the SAW group was best predicted by lower pain intensity, longer duration of pain, better pain acceptance, lower perceived physical workload, better mental health, and more psychological distress. Contrary to expectations based on the univariate analyses, higher psychological distress was (minimally) associated with being in the SAW group. In all the three domains of physical-, psychological- and work characteristics were variables that contributed to distinguish both groups, suggesting that factors from multiple domains are important for sustained work participation. Future research concerning disability prevention may target these variables that may be promising for sustained work participation. Pain related variables were strongly associated with group status, suggesting that pain intensity matters in sustained work participation. The SAW group reported on average 1.5 points less pain compared to the SL-Rehab group, which was a significant difference, but not clinically relevant [35, 91, 92]. In our study pain intensity was one of the variables that explained group membership. We do not know whether pain reduction would be effective to improve workability. Some studies concluded that disability level rises gradually with pain intensity [78, 93–95]. In other studies pain intensity was not observed as a significant predictor for work ability [16, 20, 85]. Whether pain reduction should be a target in multidisciplinary rehabilitation for CMP to improve workability is under debate. Nevertheless, workers in the SAW group have shown that sustained work participation with CMP is indeed possible.

### Strengths and Limitations of the Study

The current study is the first that provides a profile of workers with CMP who succeed to stay at work despite pain, which complemented our view on work participation in CMP and may contribute to a better understanding of work participation in non-clinical samples. People who stay at work are less accessible for research, yet we managed to include 119 participants. When group size is large, differences between groups turn out to be significant very soon, sometimes even when differences are negligible. We expressed the magnitude of the differences in ES to elevate the robustness of the results. All participants in our study were physically examined and medical data were available, so diagnoses were not solely based on self-report.

A few limitations in our study need careful attention. Participants in the SAW group responded to a call in a newspaper in which they were invited to take part in the study. In this design selection bias is inevitable and diminishes the external validity of the results. Higher educated or older workers may have been more prone to participate into the study and workers with high decision latitude had better opportunity to leave their job for a few hours and participate into the study. In our analysis we adjusted for educational level and other potential confounding variables. In this explorative study, data of the SAW and SL-Rehab group was collected at one point in time. Because of the cross-sectional data collection, no causal inferences could be made. Secondly, workers who managed to stay at work may have become sick-listed after participating into our study, thus violate the SAW condition we defined. We included workers without sick-leave during the past 12 months due to CMP. Most participants had positive expectations to remain at work the next 2 years, 20% was unsure and 1% did not expect to work after 2 years. Therefore, we considered it was not likely that many workers in the SAW group became sick-listed soon after participation into our study. We investigated workers with CMP, which was not defined as a uniform diagnosis group, and therefore might influence interpretation of data. We made this choice because in daily practice clinicians are confronted with patients who present a diversity of diagnoses with often more than one pain site [5, 96, 97]. In testing our hypotheses we controlled for diagnose group, which did not alter the results.

This study was conducted in The Netherlands. In other societies or cultures, with different compensation systems for work disability, determinants for sustained work participation may be different [98]. Our study was explorative and may be used to direct future research and clinical developments in vocational rehabilitation and sustained work participation of workers with CMP. Clinicians may use the characteristics of the SAW and SL-Rehab group to estimate the relevance of “deviant” scores of their patients. Longitudinal studies on SAW are needed to further increase our knowledge about staying at work with CMP.

## Conclusions

A wide range of bio-psycho-social characteristics of workers who stay at work despite CMP were explored. People who stay at work despite pain have clinically relevant different scores compared to sick-listed workers with CMP referred for multidisciplinary rehabilitation on fear avoidance beliefs about physical activity, pain catastrophizing, pain acceptance, pain self efficacy, life control and perceived physical workload. Group status was not associated with activity level, coping strategy and work satisfaction. The SAW and SL-Rehab group could be discriminated the best by pain intensity, duration of pain, pain acceptance, perceived physical workload, mental health, and psychological distress. Further research on these topics is needed to raise our understanding of staying at work despite CMP and to investigate the usefulness for sustained work participation.
